# Exploring awareness and perceptions of palliative care: a descriptive cross-sectional survey study in Central Europe

**DOI:** 10.3325/cmj.2025.66.27

**Published:** 2025-02

**Authors:** Ulrike Spary-Kainz, Nicole Posch, Andrea Siebenhofer, Zlata Ožvačić Adžić, Erika Zelko

**Affiliations:** 1 Institute of General Practice and Evidence-Based Health Services Research, Medical University of Graz, Graz, Austria; 2 Department of Family Medicine, University of Zagreb School of Medicine, Zagreb, Croatia; 3 Health Center Zagreb-Centar, Zagreb, Croatia; 4 Institute of General Practice, Johannes Kepler University Linz, The Life Science Park, Linz, Austria

## Abstract

**Aim:**

To assess awareness, knowledge, and preferences regarding palliative care in two Central European countries (Slovenia and Croatia) and the Austrian federal state of Styria. The study explored differences in the sources of information, public perceptions, and preferred settings for end-of-life care.

**Methods:**

In this descriptive cross-sectional survey, we enrolled a community-based sample of adults (≥18 years) residing in the three regions. Propensity score matching was applied to balance demographic variables in the analysis. Overall and matched samples were reported for the three regions.

**Results:**

The study enrolled 1586 respondents; 78.2% had at least heard of palliative care. In the matched sample (n = 462), respondents from Austria had the highest awareness of palliative care goals (74.4%), and those from Slovenia had the highest preference for obtaining information from traditional media (54.4%). Opinions on death discussions varied significantly; in Austria, there was the highest percentage (69.5%) of respondents who felt death was insufficiently discussed in society. The preferred end-of-life care setting was home, with Austrians having the highest preference for this setting (70.8%).

**Conclusion:**

The study revealed notable disparities in awareness, knowledge, and preferences regarding palliative care, although the sample size varied between countries. These differences should be addressed by tailored communication strategies and public health campaigns, which should align health care services with the preferences and needs of the population. The findings provide insights into how to improve end-of-life care and enhance public understanding of palliative services in three Central European regions.

The awareness and knowledge of palliative care vary considerably between countries. Over 80% of respondents from Northern Ireland, for example, had heard of palliative care, but 75% knew little or nothing about it (1), while only 32% of respondents from Turkey were aware of it at all (2). In the USA, less than 30% of respondents had any knowledge of palliative care (3). In Slovenia, women, older people, and highly educated respondents were more aware of palliative care (4). In Austria, 70.3% of respondents had at least heard of palliative medicine (5).

Palliative care is mostly available in Europe (6,7). Although many European countries offer good palliative care services, their availability still varies from country to country. According to Clark et al, Austria’s palliative care is in a preliminary stage of integration, Slovenia offers generalized palliative care, and Croatia offers isolated palliative care (7).

This study was conducted in two Central European countries (Croatia and Slovenia) and the Austrian federal state of Styria. While palliative care in these countries was established at about the same time, implementation strategies varied, and the number of provided palliative services differs considerably. Styria established its first facility in 1998 and currently features six palliative care units, ten counseling services, nine mobile teams, and various hospice options (8). The first palliative care team in Slovenia was established in 2000. Slovenia prioritizes accessibility and has since 2011 deployed eight mobile teams throughout the country. In addition, the Hospice House and a pediatric palliative care team have been established. These efforts increased the bed capacity to 180 by 2008 (9,10). Croatia initiated palliative care in the early 1990s, introduced the first mobile teams in 2008, and established a coordination center in 2012. As of 2020, County Coordination Centers exist in 17 of the 21 counties, offering 88 palliative care beds per million residents. In 2022, Croatia had 398 designated palliative care beds in 29 facilities (11-13).

This survey evaluated the levels of awareness and knowledge of palliative care in three Central European regions, identified the sources of information, respondents' views on societal discussions of death, and preferred settings for end-of-life care. The results provide an overview of the current state of palliative care in each country and a comparison between the countries.

## Respondents and methods

### Study design

We conducted a descriptive cross-sectional survey to assess the public awareness and knowledge of palliative care in Styria (a federal state of Austria), Slovenia, and Croatia. The respondents were asked whether the subject of death was discussed enough in society and where they would prefer to spend the final stage of their lives. The sources of their information were also identified.

The ethical approval was granted by the ethics committees of the Medical University of Graz, the Medical Faculty Maribor, and the Health Center Zagreb-Centar.

### Respondents

The respondents were a community-based sample of adults (≥18 years) residing in Styria, Slovenia, or Croatia. They were required to understand their national language, and in Austria had to sign a written consent form. In Croatia and Slovenia, the respondents who chose the paper version of the questionnaire signed a written consent form, while those who chose the online version provided their consent on the first page of the online questionnaire.

### Recruitment and data collection

Respondents were recruited from offices of doctors of different specialties. In Austria and Slovenia, an anonymous survey was conducted from October 2019 to March 2020, and in Croatia from October 2019 to October 2020. The respondents completed the study in one sitting lasting approximately 15 minutes.

A questionnaire comprising 18 questions in multiple-choice and single-choice formats was used. The questionnaire was based on the Slovenian version (4). The English version of the questionnaire is available in the Supplemental Material.(Supplementary Material)

### Statistical analyses

Only categorical variables were used, so the results are presented as frequencies and percentages. Propensity score matching was used to balance different samples in line with demographic data. A χ^2^ test was used to compare the countries in terms of awareness and knowledge of palliative care. A *P* value below 0.05 was considered significant. Statistical analysis was conducted with SPSS, version 29 (IBM Corp., Armonk, NY, USA).

## Results

### Description of the sample

In total, 1586 questionnaires were analyzed: 419 from Styria, 154 from Croatia, and 1013 from Slovenia. To account for different sizes of the country samples, we separately presented the results for the total sample and for the matched sample. A sample of 154 persons from each region was matched in terms of gender, age, and education.

In the matched sample, 5.2% of respondents were aged from 18 to 34 years, 41.6% from 35 to 49 years, 37.0% from 50 to 64 years, and 16.2% were older than 64. Furthermore, 1.9% had completed primary school and 39.0% had a university or college degree (Table 1).

**Table 1:** Demographic data by country

	Total sample (%)				Matched sample (%)			
	Austria n = 419	Croatia n = 154	Slovenia n = 1013	p*	Austria n = 154	Croatia n = 154	Slovenia n = 154	p*
Gender				0.029				0.710
female	58.2	62.3	65.6		64.9	62.3	60.4	
male	41.8	37.7	34.4		35.1	37.7	39.6	
Age (years)				<0.001				1.000
18-34	28.6	5.2	23.6		5.2	5.2	5.2	
35-49	23.6	41.6	36.5		41.6	41.6	41.6	
50-64	24.6	37.0	35.5		37.0	37.0	37.0	
65+	23.2	16.2	5.3		16.2	16.2	16.2	
Education				<0.001				1.000
without or primary school	8.4	1.9	4.8		1.9	1.9	1.9	
vocational school	28.2	17.5	8.3		17.5	17.5	17.5	
secondary school	16.7	24.7	19.1		24.7	24.7	24.7	
high school	18.1	16.9	12.9		16.9	16.9	16.9	
college or university	28.6	39.0	54.9		39.0	39.0	39.0	

*χ^2^ test.

### Awareness and knowledge of palliative medicine

Overall, 78.2% of respondents from the total sample had at least heard of palliative care. A total of 58.1% (n = 921) were somewhat aware of palliative medicine, 21.8% (n = 346) had never heard of it, 18.1% (n = 287) were well aware of it, and 2% (n = 32) were very aware of it. The results varied between countries: 29.6% of respondents from Austria, 20.4% from Slovenia, and 9.7% from Croatia had never heard of palliative care (*P* < 0.001).

In the matched sample (n = 462), 82.5% of the respondents had at least heard of palliative care. In Austria, 24.0% had never heard of palliative medicine, compared with 18.8% in Slovenia and 9.7% in Croatia (*P* < 0.001) (Table 2).

**Table 2:** Awareness of palliative care by country in the total sample and matched sample

	Total sample (%)				Matched sample (%)			
How do you rate your knowledge and awareness of palliative care?	total n = 1586	Austria n = 419	Croatia n = 154	Slovenia n = 1013	total n = 462	Austria n = 154	Croatia n = 154	Slovenia n = 154
**I know nothing about it**	**21.8**	**29.6**	**9.7**	**20.4**	**17.5**	**24.0**	**9.7**	**18.8**
**I have heard about it**	**58.1**	**55.8**	**60.4**	**58.6**	**62.1**	**63.0**	**60.4**	**63.0**
**I know a fair amount about it**	**18.1**	**11.2**	**26.0**	**19.7**	**16.9**	**9.1**	**26.0**	**15.6**
**I know a great deal about it**	**2.0**	**3.3**	**3.9**	**1.2**	**3.5**	**3.9**	**3.9**	**2.6**
		**χ^2^ = 50.929, *P* < 0.001**				**χ^2^ = 23.027, *P* < 0.001**		

**Figure 1:**
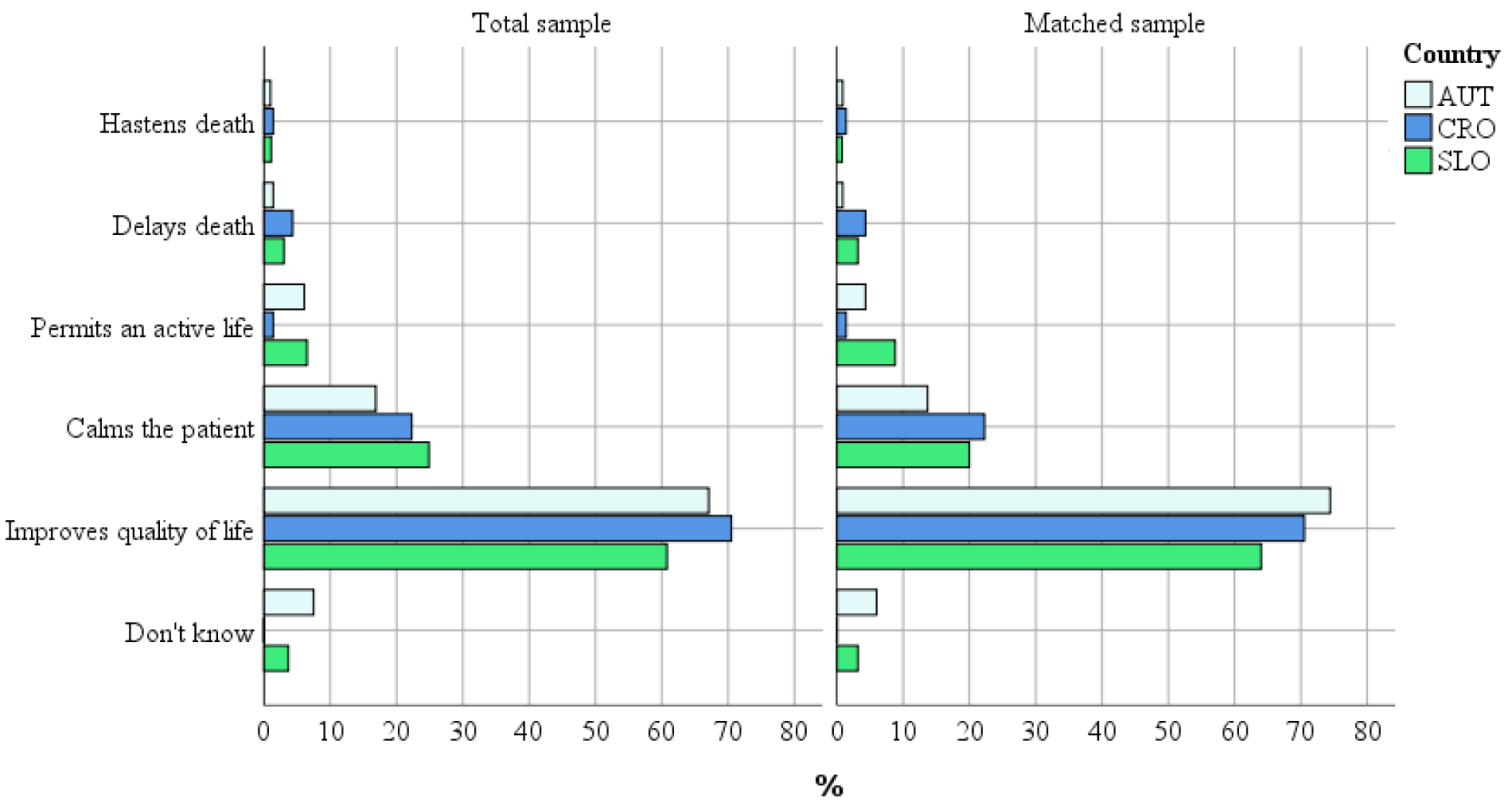
The main goal of palliative care in the total and matched samples, as reported by respondents (χ^2^ = 31.266, *P* < 0.001 and χ^2^ = 22.160, *P* = 0.014, respectively).

Only the respondents who had at least heard of palliative medicine (n = 381 in the matched sample) were asked about the main goal of palliative care (to improve the quality of life). In the total sample, 70.5% of Croatians, 67.1% of Austrians, and 60.8% of Slovenians (*P* < 0.001) knew what the main goal was. In the matched sample, this question was answered correctly by 69.6% of respondents: 74.4% in Austria, 70.5% in Croatia, and 64.0% in Slovenia (*P* = 0.014) (Figure 1).

### Sources of information

**Figure 2:**
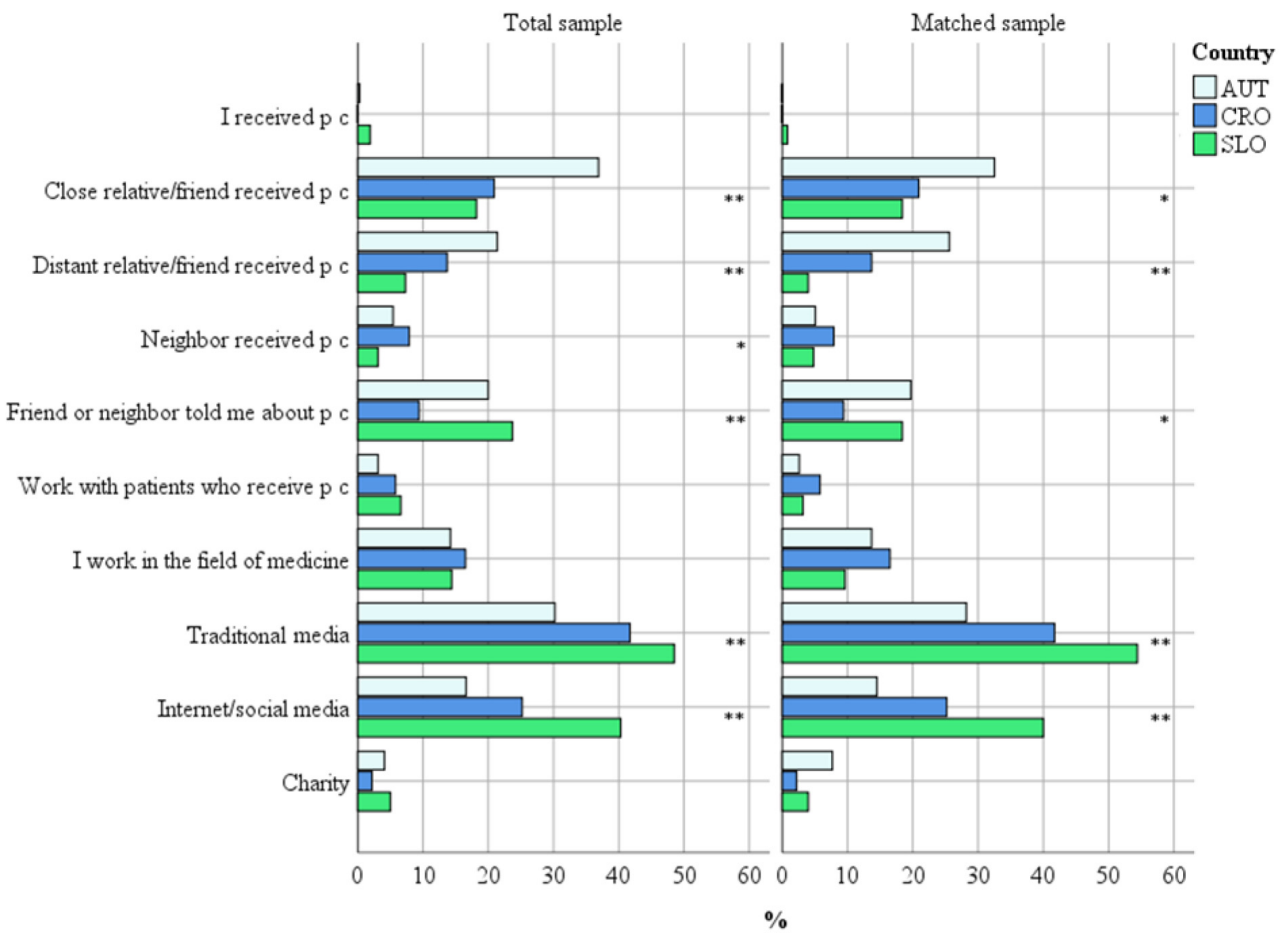
Source of information on palliative care by country in the total and matched samples, as reported by the respondents. * *P* < 0.05; ** *P* < 0.001. p.c. – palliative care.

There were significant differences between the countries in the sources of respondents’ information about palliative medicine. In the matched sample, more Austrians than respondents from the other countries had heard about palliative care from close (32.5%) or distant (25.6%) relatives, or friends who had received palliative care, followed by their friends and neighbors (19.7%). In Slovenia, more people said their information had come from traditional media channels (54.4%) or internet/social media (40%) than in Croatia (41.7% and 25.2%, respectively) and Austria (28.2% and 14.5%, respectively). The total sample showed the same trend. Several answers to this question were possible (Figure 2).

### Societal discussion of death

On average, 66.6% of respondents in the total sample felt that the topic of death was not discussed sufficiently in society. However, there were significant differences between countries (*P* < 0.001). Overall, 68.7% of Slovenian respondents, 67.3% of Austrian respondents, and only 50.6% of Croatian respondents felt that death was discussed insufficiently.

In the matched sample, 61.5% respondents thought that death was discussed insufficiently. However, significant differences existed between the countries (*P* = 0.005). In Austria, 69.5% of respondents felt that death was discussed insufficiently, while only 2.6% thought it was discussed sufficiently. The respective percentages in Slovenia were 64.3% and 6.5%, and in Croatia – 50.6% and 9.7% (Table 3).

**Table 3:** Respondents’ perception on whether death and dying are sufficiently discussed in society

	Total sample (%)				Matched sample (%)			
How much do you think people generally speak about death and dying?	total n = 1586	Austria n = 419	Croatia n = 154	Slovenia n = 1013	total n = 462	Austria n = 154	Croatia n = 154	Slovenia n = 154
Not enough	66.6	67.3	50.6	68.7	61.5	69.5	50.6	64.3
About enough	29.7	29.4	39.6	28.3	32.3	27.9	39.6	29.2
Too much	3.7	3.3	9.7	3.0	6.3	2.6	9.7	6.5
		χ^2^ = 29.071, *P* < 0.001				χ^2^ = 14.935, *P* = 0.005		

### The preferred setting for end-of-life care

In the total sample, on average 70.1% of respondents wanted to spend the final stages of their lives at home: 74.7% in Austria, 69.6% in Slovenia, and 60.4% in Croatia. In the matched sample, home was the preferred end-of-life setting for 70.8% of respondents from Austria, 66.9% from Slovenia, and 60.4% from Croatia. Correspondingly, 4.5% of Austrian respondents, 7.8% of Slovenian respondents, and 15.6% of Croatian respondents preferred a hospital. Finally, 3.9% of Austrian, 5.2% of Croatian, and 3.9% of Slovenian respondents preferred a nursing home (Table 4).

**Table 4:** Respondents’ answers on the preferred setting for end-of-life care

	Total sample (%)				Matched sample (%)			
If you had a terminal illness, where would you like to spend the final stage of your life?	total n = 1586	Austria n = 419	Croatia n = 154	Slovenia n = 1013	total n = 462	Austria n = 154	Croatia n = 154	Slovenia n = 154
Home	70.1	74.7	60.4	69.6	66.0	70.8	60.4	66.9
Hospital	7.3	3.8	15.6	7.5	9.3	4.5	15.6	7.8
Nursing home	3.8	4.3	5.2	3.5	4.3	3.9	5.2	3.9
Don’t know	18.8	17.2	18.8	19.4	20.3	20.8	18.8	21.4
		χ^2^ = 26.986, *P* < 0.001				χ^2^ = 12.613, *P* = 0.050		

## Discussion

In this study, the awareness and knowledge of palliative care differed between the three Central European regions. Only 9.7% of respondents from Croatia had never heard of palliative care, compared with 18.8% in Slovenia and 24.0% in Austria. Such differences between similarly well-developed countries may be explained by variations in health care systems and strategies employed to implement palliative care. Croatia implemented a strategic system-level intervention from 2014 to 2020 to introduce a new integrated palliative care model (12). It seems that the intervention was far-reaching as only about 9% of the respondents in our survey said they knew nothing about palliative care.

In our study, respondents from different countries had different opinions on whether death was sufficiently discussed in society. In Austria, 69.5% of respondents in the matched sample felt death was not discussed enough, while only 2.6% felt it was discussed too often. In Croatia, only 50.6% felt it was not discussed enough, and 9.7% thought it was discussed too often. In a study by the Croatian Coalition of Associations in Healthcare, 76.3% of respondents were willing to answer questions related to death and dying, and 41.7% of respondents strongly agreed that talking about death and dying was something that people did not like (14). If palliative care is to become acceptable both near the end of life and in earlier stages of a terminal illness, health care providers and the general public should feel comfortable to speak freely about the topic. For this reason, the topic should also be included in the medical curriculum.

Overall, 70.8% of respondents from Austria, 66.9% from Slovenia, and only 60.4% from Croatia preferred their home as the setting for end-of-life treatment. Contrary to many people’s desire to die at home, in Austria in 2019, 48.9% of people died in hospital, 25.2% at home, and 20.1% in a nursing home (15). In Croatia in 2022, the rates were 50.6%, 28.8%, and 17.7%, respectively (16). In Slovenia, 48.4% died in hospital and 44.7% at home or in a nursing home. Data on Slovenian respondents did not distinguish between dying at home or in a nursing home (17). These findings show that the desire of most people to die at home does not correspond to reality, as has also been revealed in several international studies (18-20). Gomes identified several conditions that should be fulfilled if patients are to die at home rather than in a hospital: it must be the patient's and the caregiver’s wish, and palliative care and district or community nursing must be available at home (19). To enable more people to die at home and still have their needs met, programs such as the Last Aid Course encourage citizens to become involved in home care (21,22). According to Kellehear (23), not only families and health services, but also communities, are responsible for end-of-life care. Public campaigns on this topic could help in raising the awareness of this issue.

It is difficult to explain why the wish to die at home was stronger in Austria and Slovenia than in Croatia. One explanation may be that in Croatia, the culture of volunteering is still underdeveloped, as opposed to Austria, where civil society plays a key role in care provision (12). Therefore, Croatian respondents may believe that end-of-life care for their relatives or friends is not their responsibility but the responsibility of the state. In Slovenia, long-term home care is still difficult to organize. As a result, fewer terminally ill patients die at home (4).

In order to strengthen palliative care, it is necessary to consider the social aspects of the target population in each country and develop campaigns that reach all communities. Unfortunately, there is no one-size-fits-all solution for the western world as attitudes toward death are not uniform (24). However, even hard-to-reach target groups have an interest in seeking information in order to understand their health problems (25). There is a need to disseminate knowledge throughout society and to minimize the taboo surrounding dying (26).

The main goals of palliative care are supporting patients and their families at the end of life and maintaining quality of life (27). This is why the idea behind palliative care should be communicated to lay people. Only when people feel they have the right to decide where and how they want to die will it be possible to deliver palliative care to the entire society and empower people to decide for themselves. It is people’s right and duty to ask for resources that can help them and their families in end-of-life care, such as mobile palliative care teams.

A possible limitation of the current study is that in Austria, only hard copies of the questionnaire were used, while in Slovenia and Croatia, both paper and online versions were utilized. This may have resulted in the recruiting process being less inclusive in Austria, as individuals who preferred or were only able to participate online were not reached. Another limitation is that all but one of the questions were closed-ended, preventing the respondents from giving additional responses besides the provided ones. However, we were not able to change the structure of the questionnaire because we wanted to ensure the international comparability of the results. As the numbers of respondents varied substantially between the countries, we used matched samples for better comparability. Nonetheless, the results in both matched and total samples were similar. However, with 154 people per country, the matched sample was relatively small. The sample cannot therefore be considered representative of the countries as a whole.

Despite its limitations, the study reveals regional differences in terms of awareness, knowledge, and preferences for palliative care. These differences point to varying needs and challenges in different regions. Although our results cannot be generalized to the entire Central Europe, the study can serve as a starting point for more targeted, comprehensive studies. The survey was also carried out in Hungary, but the results were not included in this analysis due to a translation error in the questionnaire.

The study provides insights into regional differences in the perception and understanding of palliative care, which makes it a useful asset for the planning of future research projects, both at the regional and supra-regional level. The findings also indicate that effective measures to improve palliative care vary across regions and require a careful needs analysis. Despite its limitations, the study contributes to a better understanding of the complex landscape of palliative care and emphasizes the need for differentiated, region-specific approaches in research and practice.
