# Supplementary material for: Exploring awareness and perceptions of palliative care: a descriptive cross-sectional survey study in Central Europe
Source: Croat Med J. 2025 Feb;66(1):27–34. doi: 10.3325/cmj.2025.66.27 (PMC11947979; doi:10.3325/cmj.2025.66.27)
Supplement: Supplementary Material [file CroatMedJ_66_s002.pdf]

QUESTIONNAIRE ON THE AWARENESS AND KNOWLEDGE OF PALLIATIVE MEDICAL CARE

1. In your opinion, what are the greatest fears of a patient who has been diagnosed with a terminal illness? (Please select three of the following options and rank them in order of relevance – 1st, 2nd, 3rd)

___ Fear of pain.

___ Fear of death.

___ Fear of no longer being independent.

___ Fear of losing mental capabilities.

___ Fear of loneliness.

___ Fear of becoming disabled/immobile.

___ Fear of financial burdens.

___ Fear of being a burden to someone.

___ I don’t know.

2. What services do you think should be provided to people with a terminal illness and their families? (You may give several answers.)

a) Help at home in case of pain and other symptoms.

b) Domestic care for the patient during the day.

c) Domestic care for the patient at night.

d) Complementary therapies.

e) Pastoral care.

f) A social worker that the patient and family could reach on the phone if necessary.

g) Assistance with household tasks, shopping, transportation etc.

h) Additional support at home to allow the family caregivers to have some free time and time for themselves.

i) Information and advice on financial aid.

j) Medical care in a hospice.

k) Medical care in hospital.

3. How much do you think people generally speak about death and dying? (Please select one answer only.)

a) Too little.

b) About the right amount.

c) Too much.

4. If you had a terminal illness, where would you like to spend the final stage of your life? (Please select one answer only.)

a) At home.

b) In a hospital.

c) In a nursing home.

d) I don’t know.

5. Do you know what a patient decree is?

a) Yes.

b) No.

6. How do you rate your knowledge and awareness of palliative care? (Please select one answer only.)

a) I know nothing about palliative care.

(If "a" is true for you, please continue to question 15.)

b) I have heard about palliative care.

c) I know a fair amount about palliative care.

d) I know a great deal about palliative care.

7. If you have already heard of palliative care, where did you find out about it? (You may give several answers.)

a) I have received palliative care myself.

b) A close friend/relative has received palliative care.

c) A distant relative/acquaintance has received palliative care.

d) A neighbor has received palliative care.

e) A friend or relative has told me about it.

f) I work with patients that receive palliative care.

g) I work in the field of medicine.

h) Radio/TV/newspaper.

i) Internet/social media.

j) Fundraising/charity drives.

k) Other: ______________________________

8. Please select the following sentence that in your opinion best describes palliative care. (Please select one answer only.)

a) Palliative care hastens death.

b) Palliative care delays death.

c) Palliative care permits the patient to continue living an active life.

d) Palliative care calms the patient.

e) Palliative care improves the patient's quality of life.

f) I don't know.

9. What do you think are the greatest needs of patients with a terminal illness that have reached the end of their lives? (Please select three of the following options and rank them in order of relevance – 1st, 2nd, 3rd)

____ Reduction in physical suffering.

____ Specialist medical care.

____ Home nursing care (medical and nursing services).

____ Support for home-based caregivers.

____ Professional psychological support.

____ Spiritual support.

____ I don't know.

10. What do you think are the greatest needs of family members that provide care to a patient with a terminal illness? (Please select three of the following options and rank them in order of relevance – 1st, 2nd, 3rd)

____ 24-hour specialist care.

____ Home nursing care.

____ Access to and availability of voluntary caregivers.

____ Specially adapted residential facilities/hospices.

____ Psychological care.

____ Grief counseling.

____ I don't know.

11. Where and by whom do you think care is best provided to patients with a terminal illness? (Please select one answer only.)

a) At home by family/relatives.

b) At home by professional care workers.

c) In a hospice.

d) In hospital.

e) I don't know.

12. Has anyone in your family or circle of friends and acquaintances had any personal experience of palliative care? (Please select one answer only.)

a) Yes.

b) No.

13. Among patients with the illnesses listed in the table below, which do you think should receive palliative care? (Please mark one of the options for each illness.)

	5 (I fully agree.)	4 (I agree.)	3 (I partly agree.)	2 (I don’t agree.)	1 (I don’t agree at all.)	I don’t know	I don’t know anything about this illness
Cancer							
Kidney failure							
Amyotrophic Lateral Sclerosis (ALS)							
Dementia							
Stroke							
Heart failure							
HIV/AIDS							
Chronic lung diseases							
Multiple Sclerosis							

14. If you were diagnosed with a terminal illness, when would you like to be provided with information on palliative care? (Please select one answer only.)

a) I would not want any information.

b) I would like the information to be publicly available.

c) I would like to receive such information when my terminal illness became life-threatening.

d) I would like to receive such information when it became clear that I was dying of my terminal illness.

15. How old are you? __

16. Gender:

a) Female

b) Male

17. Highest level of education:

a) Compulsory schooling incomplete

b) Compulsory schooling

c) Apprenticeship

d) Technical college/trade school

e) High school (general qualification for university entrance)

f) University of Applied Sciences

g) University

18. Postal code of your home: ________________
